# From Husks and Seeds to Health: an Inevitable Outcome Rather than a Fluke

**DOI:** 10.1007/s13668-025-00722-4

**Published:** 2026-02-26

**Authors:** Nevin Sanlier, Ebru Ozler

**Affiliations:** Department of Nutrition and Dietetics, School of Health Sciences, Ankara Medipol University, Altındağ, Ankara, 06050 Turkey

**Keywords:** Psyllium, Dietary fiber, Obesity, Diabetes, Constipation

## Abstract

**Purpose of Review:**

This review was conducted to examine the promising health benefits of psyllium, its therapeutic potential in relation to various chronic diseases, its mechanisms, and its possible side effects or toxicity; to explore its applications in food systems; to identify the positive and negative effects of its consumption; and to provide perspectives for future research and development efforts for psyllium.

**Recent Findings:**

Psyllium, also known as ispaghula, is a dietary fiber obtained from the shells of *Plantago ovata* seeds. The most abundant monosaccharides in its composition are xylose and arabinose. Psyllium is a natural alternative to the use of food additives such as gum and hydrocolloid to increase the fiber content in fortified foods. Psyllium may have therapeutic effects on various diseases. In recent years, its potential in the management of conditions such as hypertension, diabetes, and liver disease has been emphasized and multifaceted therapeutic applications have been introduced. Psyllium may help to regulate blood glucose, blood pressure and cholesterol levels. It may also increase feelings of fullness and reduce food intake. Furthermore, psyllium may promote bowel regularity and improve the composition of gut microbiota. Psyllium has been reported to exhibit antioxidant and antimicrobial properties, as well as reducing inflammatory mediators such as TNF-α and nitric oxide. Furthermore, psyllium has been associated with lower creatinine and uric acid levels, as well as a reduced risk of carcinogenesis.

**Summary:**

Studies in the literature support the therapeutic effects of psyllium. However, longer-term clinical trials are needed to determine the effective dose and duration of use. Innovative product development studies should be conducted to enhance the functional benefits of psyllium in the food industry.

**Supplementary Information:**

The online version contains supplementary material available at 10.1007/s13668-025-00722-4.

## Introduction

The shells or husks of *Plantago ovata* seeds, also known as psyllium, ispaghula, or isabgol, are attracting attention in recent years due to their health benefits [1, 2]. This plant is native to the Mediterranean region and also frequently grown in India, Iran, and Pakistan [3]. One of the main nutritional properties of psyllium husk is its high fiber content [4, 5].

Psyllium is used as a hydrocolloid in the food industry thanks to its water absorption capabilities [6]. It is used to preserve moisture and extend the shelf life of bakery products [7], as a fat substitute in milk and dairy products [8], as a binder in meat and meat products, to increase the fiber contents of breakfast cereals, and to provide a feeling of satiety in meal replacement shakes and nutritional bars [1].

Psyllium may have beneficial impacts on many health problems. In particular, it may be effective in the management of conditions such as hypertension, diabetes, and liver disease, alongside other potential health benefits, such as its ability to support gastrointestinal health, cardiovascular risk reduction, and metabolic control. It has a therapeutic effect against diabetes by regulating blood glucose, against obesity by reducing energy intake, against cardiovascular diseases by reducing blood pressure and cholesterol levels, and against gastrointestinal system diseases by regulating defecation [9, 10].

In recent years, there has been an increase in the number of studies in the literature highlighting the health effects of psyllium. New publications support the beneficial effects of psyllium on lipid metabolism, blood pressure, and glycemic control. Studies also emphasize its various metabolic and hepatoprotective effects, including the modulation of bile acid metabolism and the activation of pathways associated with the farnesoid X receptor. Therefore, updated reviews are needed to integrate these new findings and comprehensively address the health effects of psyllium.

This review study was conducted to examine the clinical effects and modes of action of psyllium in its relationships with many diseases, as well as its positive and negative effects on health as revealed by in vivo, in vitro, animal, and human studies. Furthermore, suggestions are made for future research.

## Methods

This article is a narrative review that examines the effects of psyllium on health. A comprehensive literature review was conducted using the PubMed, Web of Science, Scopus, and Google Scholar databases. Articles published in English between January 2018 and January 2025 were evaluated. The main search terms included combinations of the following keywords: “psyllium,” “psyllium husk,” “*Plantago ov*ata,” “dietary fiber,” “obesity,” “diabetes,” “cardiovascular diseases,” “diarrhea,” “constipation,” “ulcer,” “non-alcoholic fatty liver disease,” “gastritis,” “gastroesophageal reflux,” “irritable bowel syndrome,” “inflammatory bowel diseases,” “cancer,” “kidney diseases,” and “burn.” English-language research articles, systematic reviews, meta-analyses, review articles, clinical human and animal studies, and cell-based research studies were subsequently analyzed. The obtained English-language documents were classified as original research, reviews, meta-analyses, or systematic reviews after being obtained in full-text format. Studies that did not include psyllium were excluded. Since this study is not a systematic review, a formal PRISMA-based screening process was not conducted. The abstracts and full texts of relevant articles in the literature were examined. All analyzed studies were accessed using the databases and keywords specified here. For the years 2017–2025, a total of 143 sources were examined and 15 highlighted articles are listed in Table 2. Studies conducted before 2017 were not included in the present review, as their findings have already been discussed in previously published reviews. However, several key references were cited where necessary to provide historical context.

### Nutrient Composition of Psyllium

Psyllium husk comprises 84.98% carbohydrates, 6.83% protein, and 4.07% ash [1], while psyllium seed flour contains 78.88% carbohydrates, 13.3% protein, 0.38% fat, and 5.0% ash [11]. Thus, psyllium husk has lower protein and fat contents than psyllium seeds, while its levels of carbohydrates and dietary fiber are higher [12]. The nutrient composition of its seeds consists largely of carbohydrates [9] and the ratio of insoluble fiber to soluble fiber is 30/70 [13]. The polysaccharide fraction of psyllium husk is an important functional component [4, 5]. The polysaccharide that forms mucilage is a heteroxylan consisting of (1→4)-linked β-D-xylose with C-2 or C-3 side chains and containing different proportions of xylose and arabinose [4]. In addition, its potassium, phosphorus, and sodium contents are higher compared to other minerals [9]. Psyllium seeds and husks also contain bioactive components such as flavonoids and polyphenols [13]. Table 1 provides detailed information about the nutritional composition of psyllium husk [14–16].

**Table 1:** Nutritional composition of psyllium husk and psyllium seed [14–16]

Nutritional components	Psyllium husk	Psyllium seed
Moisture (%)	4.9	1.91
Ash (%)	4.1	1.0
Crude fat (%)	1.2	3.75
Crude protein (%)	3.9	17.70
Nitrogen free extract (%)	78.2	-
Total dietary fibre (%)	77.24	24.77
Arabinose (%)	46.8	-
Xylose (%)	24.1	-
Glucose (%)	11.2	1.89
Galactose (%)	2.09	-
Mannose (%)	4.24	-
Rhamnose (%)	2.39	-
Calcium (mg/100 g)	104	-
Potassium (mg/100 g)	805	687
Sodium (mg/100 g)	62.3	-
Iron (mg/100 g)	9.3	6.75
Copper (mg/100 g)	0.36	2.39
Manganese (mg/100 g)	1.15	1.06
Zinc (mg/100 g)	ND	3.15

ND: not detected

### Bioavailability and Mechanisms of Action of Psyllium

Since psyllium is not fermented in the intestine, its structure and its functions are not impaired in the gastrointestinal system. This increases the bioavailability and effectiveness of psyllium for various health applications. Psyllium consists of 65% insoluble and 35% soluble polysaccharides, and the hemicellulose present in psyllium contributes to its gelling and water retention capacities [17]. When psyllium is diluted, it forms a viscous gel that slows nutrient absorption, increases chyme viscosity, and increases the feeling of satiety [18]. Therefore, psyllium may support intestinal health and may have positive effects on health through various mechanisms of action, potentially reducing inflammation, helping to regulate the intestinal microbiota and its motility, and exerting anti-inflammatory effects in conditions such as irritable bowel syndrome (IBS) and colitis. Psyllium may contribute to the growth of beneficial bacteria, which may positively influence the composition of the intestinal microbiota [19]. In an animal study conducted with rats, psyllium was found to have antioxidant and anti-inflammatory effects [20]. In another animal study, psyllium was reported to significantly reduce colon damage and inflammation in mice [21]. In a food formulation study, the addition of psyllium to rye bread was found to increase the antioxidant activity of the bread by two-fold [5]. Psyllium may support intestinal health and may increase antimicrobial protein expression by neutralizing pathogenic microorganisms [22]. In an in vitro study, a nanoemulsion formed by adding *Cuminum cyminum* essential oil to *Plantago psyllium* seed gum inhibited the growth of *Staphylococcus aureus* [23]. In murine models, psyllium was found to upregulate antimicrobial proteins such as small proline-rich protein 2 A (SPRR2A) and resistin-like beta (RELMβ) in the small intestines of mice [22]. In an experimental study using in vitro assays and a rat wound model, psyllium and Carbopol-based frankincense essential oil were observed to suppress microbial growth and increase microbial death [24]. In an in vitro experimental study, psyllium-based polymeric antimicrobial agents showed antibacterial activity against gram-positive bacteria such as *Staphylococcus aureus* and *Bacillus anthracis* [25]. Psyllium may help improve constipation and may help normalize stool consistency via its capabilities for intestinal regulation [19]. It forms a viscous gel that binds bile acids, slows the absorption of cholesterol and glucose, creates a feeling of satiety, and increases the release of glucagon-like peptide-1(GLP-1). However, to fully utilize these significant health benefits, personalized approaches are needed while using psyllium as a dietary supplement due to individual differences in gut microbiota compositions and diets [22].

When examining the mechanism of action of psyllium, it is observed that the findings are generally based on preclinical evidence, including animal and in vitro studies. These studies provide valuable mechanistic hypotheses; however, the findings need to be confirmed in human clinical trials.

### Use of Psyllium in Food Products

Awareness of healthy nutrition is steadily increasing worldwide and a wide variety of functional foods are being produced for this purpose. Considering its potential health effects, psyllium may be used in the food sector in the production of items such as gluten-free breads, cakes, biscuits, pasta, noodles, pizza, and ice cream [13]. The use of psyllium in food products serves two main purposes. First, it may constitute an alternative to the use of gum and hydrocolloid as food additives, and second, it may enrich the nutritional content of food products. Psyllium has high water absorption capacity; in food production, it may contribute to product quality by reducing the water and fat contents of foods [3].

Psyllium may increase dough viscosity and gas retention during baking; may help prevent the loss of softness, moisture, and elasticity during the storage of baked goods; may improve volume; and may increase water-binding capabilities. For these reasons, it is commonly used in a wide range of bakery products. It may improve the texture and structure of gluten-free bread and may extend its shelf life. In addition, it may enrich fiber contents and may reduce the glycemic index [7]. Researchers have found that adding psyllium to gluten-free bread improves the color and textural properties of the bread, increases the gas retention capacity of the psyllium, and exerts a gluten-like effect; thus, this approach can be used in the production of bread with low glycemic index values [26]. In another study, it was reported that adding psyllium to whole wheat bread reduces hardness, increases flexibility, and increases acceptability and nutritional quality [27]. Psyllium may also be added to breakfast cereals due to its fiber contents. It may be used in meal replacement shakes because of its abilities to increase satiety, decrease appetite, and regulate body weight control. It may be used in meat products such as sausages and meatballs to help retain moisture, prevent cooking loss, maintain the texture of the product, and increase the binding capabilities [1]. It was reported that adding water and *Plantago ovata* to minced chicken increased production efficiency and reduced the hardness of the product [28]. In a study in which psyllium was added to phosphate-free meat at different rates, 1.5% psyllium husk powder was found to increase product quality [29]. Psyllium may be used as a thickener in milk to improve the consistency and texture of dairy products such as yogurt and ice cream [1]. The addition of *Plantago psyllium* mucilage to Doogh, a yogurt-based fermented product, increased viscosity, reduced phase separation, and improved the stability of the product [30]. Furthermore, adding psyllium as a fat replacer in yogurt production provided appropriate viscosity, created less syneresis, and increased the water retention capacity [8].

**Fig. 1:**
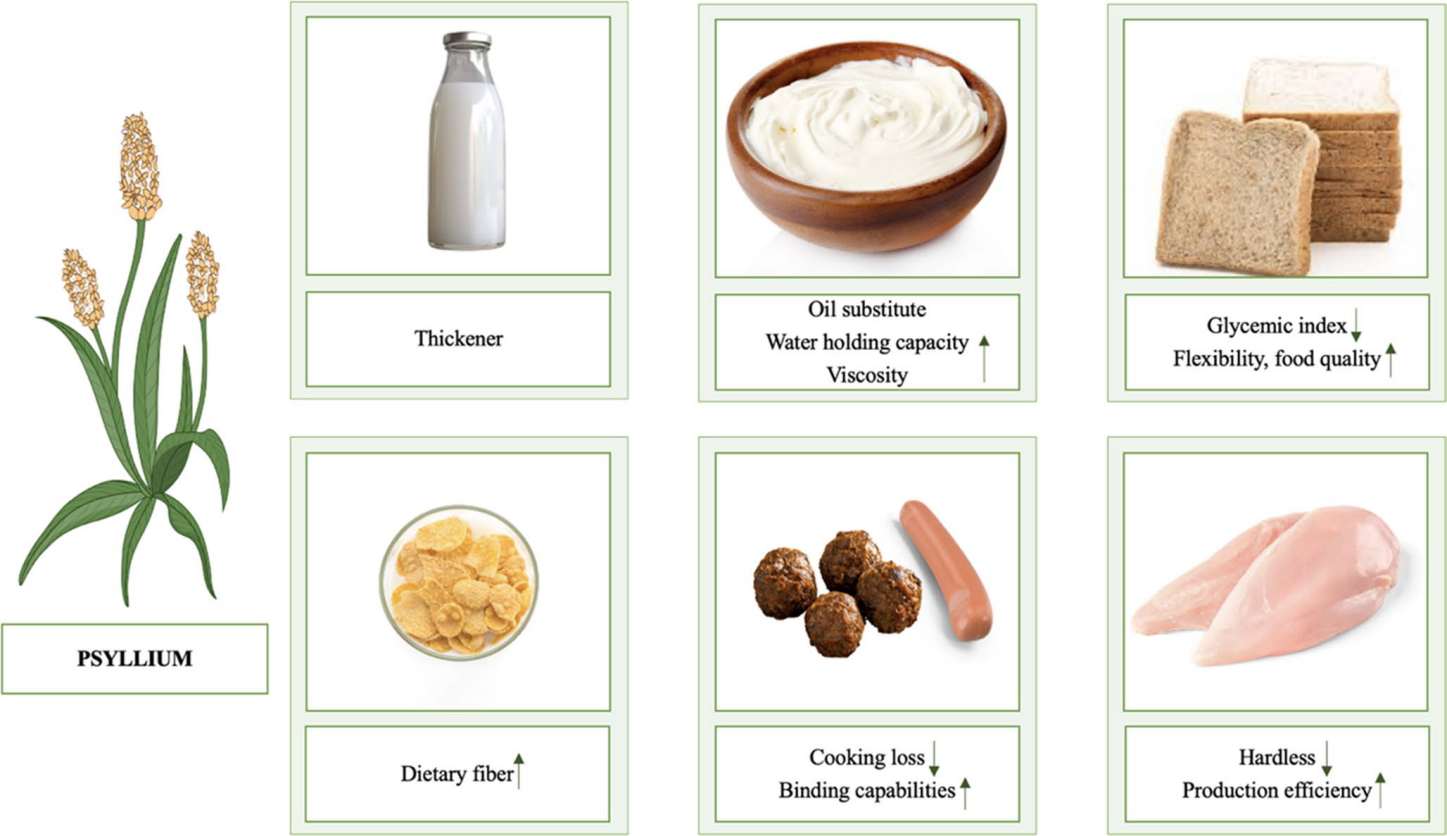
Use of psyllium in food products [1, 7, 8, 27, 28]

However, the interactions of psyllium with other macronutrients and micronutrients in foods are not fully understood. More research is needed to reduce undesirable effects in foods to which psyllium is added, such as gummy textures, and to increase psyllium’s positive effects on foods [3]. The applications of psyllium in food products are shown in Fig. 1.

### Therapeutic Effects of Psyllium

In addition to its use as a laxative to support intestinal health, psyllium has various other therapeutic applications. In recent years, it has been used in the treatment of many diseases. Psyllium may help reduce cholesterol levels and absorption by binding bile acids in the intestine [31, 32]. It may have beneficial effects for diabetic patients by providing glycemic control as a result of its soluble fiber contents. The antihypertensive properties of psyllium may positively affect cardiovascular health [13]. Psyllium may help relieve diarrhea symptoms and may exert therapeutic effects in cases of gastrointestinal disorders. Furthermore, it may increase the therapeutic potential of various drugs, including antibiotics and anticancer drugs, and may be used to form hydrogels for sustained drug delivery [33, 34]. However, alongside these positive effects in a variety of therapeutic areas, it should be taken into account that dietary fibers may cause different side effects for different individuals.

### Effects of Psyllium on Health

Psyllium has positive effects on a wide variety of health problems as it creates feelings of satiety, lowers cholesterol levels, and possesses prebiotic properties. Constipation, diarrhea, cancer, diabetes, obesity, inflammatory bowel diseases (IBD), and hypercholesterolemia are among the health problems that psyllium may positively affect [9].

### Obesity

It is predicted that approximately 20% of the female population and 14% of the male population will be obese worldwide by 2030 [35]. As a possible solution, dietary fiber is negatively associated with body weight and body mass index (BMI). Psyllium may play important roles in body weight management and the treatment of obesity-related diseases. Its gel-forming properties may support intestinal health and metabolic functions, making it a suitable dietary supplement for overweight and obese individuals [18]. Psyllium may affect body composition through mechanisms such as intestinal hormones like cholecystokinin, satiety, and gastric emptying [36]. It has been reported that psyllium may alleviate obesity-related inflammation through mechanisms independent of fermentation and intestinal microbiota interactions [37]. However, debates regarding the effect of psyllium on obesity are still ongoing [36].

A meta-analysis study found that psyllium supplementation at an average dose of 10.8 g/day before meals led to reductions in body weight (2.1 kg), BMI (0.8 kg/m^2^), and waist circumference (2.2 cm) in overweight and obese participants [18]. Another meta-analysis study concluded that there was moderate evidence that psyllium provided a small improvement in body weight loss [38]. Obese adults with genetic polymorphisms including at least one minor allele of the melanocortin 4 receptor (*MC4R*), fat mass and obesity-associated gene (*FTO*), leptin receptor (*LEPR*), and leptin (*LEP*) polymorphisms were given fiber supplements containing psyllium, glucomannan, and inulin for 180 days and the intervention was shown to achieve significant reductions in body weight, BMI, fat mass, and visceral fat ratio [39]. In another study, psyllium supplementation of 5 g three times daily (15 g/day) for 52 weeks administered to obese and overweight adults was found to have reduced zinc, sodium, folate, and magnesium intake after 3 months, which could be attributed to the satiating effect of dietary fiber and the reduction of food intake, while there was no significant difference in serum micronutrient concentrations [40]. It was also found that psyllium reduced waist circumference and BMI in obese adolescents without any side effects [41]. In adults with central obesity, the combination of psyllium husk pulp and lifestyle changes was found to reduce gastrointestinal system symptoms but did not affect sleep quality [42]. Furthermore, a supplement containing psyllium, glucomannan, inulin, and apple pulp reduced BMI, fat mass, visceral fat, and C-reactive protein levels together with energy restriction in overweight and obese individuals [43]. In a study investigating the effectiveness of an herbal formulation containing a mixture of *Plantago psyllium*, *Portulaca oleracea*, and peanut oil in overweight and obese adult individuals for 8 weeks, the product was shown to reduce body weight and suppress appetite [44]. In a single-blind randomized controlled study, patients with BMI values of 20–47 kg/m^2^ were given cookies containing 10 g of psyllium twice a day for 12 weeks and it was reported that their body weights decreased [45].

Many meta-analyses and clinical studies have reported that psyllium supplementation leads to reductions in BMI and body weight. However, some studies have shown that these effects were not statistically significant or resulted in only minimal changes. These inconsistencies among studies may be attributed to differences in study design, evaluated parameters, individual characteristics of participants, psyllium dose or form, and intervention duration. However, while it shows promise in applications for body weight management, its effects may vary between individuals. It should be considered as one part of broader lifestyle change strategies for obesity management.

### Diabetes Mellitus

Diabetes affects 537,000,000 individuals worldwide (10.5%) between the ages of 20 and 79 [46]. Lifestyle changes, nutrition, and exercise are all crucial in the management of diabetes. It is known that dietary fiber reduces the risk of diabetes, and psyllium, a type of dietary fiber, has beneficial effects in controlling blood sugar levels and improving insulin sensitivity in diabetes management. The effects of dietary fiber on glycemic control vary [47].

There are studies in the literature demonstrating that psyllium has beneficial effects on fasting blood glucose [48, 49]. However, consensus has not been reached regarding the psyllium dosage to be used for positive effects in cases of diabetes [47]. Psyllium supplementation may reduce fasting blood sugar, hemoglobin A1c (HbA1c), and homeostasis model assessment of insulin resistance (HOMA-IR) levels, and it does not significantly affect insulin levels [50]. In C57BL/KsJ db/db diabetic mice, psyllium supplementation has been reported to decrease fasting glucose level while increasing insulin levels [51]. It has also been found that psyllium slows glucose absorption and may be beneficial when added to low glycemic index foods such as fortified chapati to reduce postprandial glucose levels [52]. In a meta-analysis study, it was concluded that psyllium fiber reduces HbA1c levels [53]. Another meta-analysis study showed that psyllium supplementation reduced fasting blood sugar and HbA1c levels in diabetic individuals but did not have significant effects on BMI, body weight, or HDL-C levels [47]. In individuals with diabetes, the most effective interventions for fasting blood glucose and HOMA-IR were found to involve the administration of β-glucan and psyllium [49]. It was reported that 16 weeks of psyllium supplementation and lifestyle changes among obese adults reduced HOMA-IR and fasting blood glucose levels [48]. Furthermore, the administration of flaxseed and psyllium (10 g/day) to diabetic individuals for 12 weeks improved glycemia and lipid profiles [54]. Psyllium at 5.1 g/day or 7.7 g/day was not found to have an effect on postprandial glycemia in individuals with cystic fibrosis-associated diabetes [55]. In another study, however, it was reported that vildagliptin-loaded psyllium-alginate nanoparticles exhibited antidiabetic properties [56]. It was also reported that exercise and *Plantago psyllium* administration improved glucose tolerance in diabetic rats [57].

Metabolic syndrome is a condition characterized by metabolic irregularities such as dyslipidemia, central obesity, hypertension, and insulin resistance [58]. A meta-analysis study on the effectiveness of psyllium against metabolic syndrome reported that it reduced fasting blood glucose and systolic blood pressure while causing a nonsignificant decrease in triglyceride levels and nonsignificant increases in diastolic blood pressure, HDL-C, and waist circumference [59]. It was reported that the administration of an herbal compound (aloe vera leaf gel, 1 g/day psyllium seed, 1.8 g/day black cumin, 300/day mg garlic, 2.5 g/day fenugreek seed, and 500 mg/day milk thorn seed) once daily for 12 weeks in patients with type 2 diabetes and dyslipidemia despite the use of statin-derived drugs reduced triglyceride, total cholesterol, low-density lipoprotein cholesterol (LDL-C), and HbA1c levels but did not affect fasting blood glucose [60]. Obesity and metabolic syndrome cause inflammation in many organ tissues, including the intestines. It has been reported that psyllium reduces intestinal inflammation in obese mice fed high-fat diets [37].

Many meta-analyses and clinical studies have shown that psyllium lowers fasting blood glucose, HOMA-IR, and HbA1c levels. However, some studies have reported limited or non-significant effects on insulin level. These differences among studies may be attributed to variations in psyllium dose and type, duration of intervention, and individual characteristics of participants.

While psyllium shows promise in managing diabetes and its complications, it is important to consider individual dietary needs and possible gastrointestinal effects. Integrating psyllium into a balanced diet, possibly in conjunction with other therapeutic strategies, may optimize its benefits for patients with diabetes. It is often used in conjunction with lifestyle and dietary changes to enhance its therapeutic effects. Further research is needed to examine its long-term effects and interactions with other dietary components.

### Cardiovascular Diseases

Cardiovascular diseases are among the leading causes of death worldwide [61]. It is estimated that 35.6 million people will die globally from cardiovascular diseases, 20 million from ischemic heart diseases, and 18.9 million from high systolic blood pressure in 2050 [61, 62]. Dietary fiber exerts protective effects against cardiovascular diseases by lowering total cholesterol and LDL-C [63]. In particular, the seed coats of *Plantago ovata* may have antioxidant, anti-inflammatory, and blood lipid-lowering effects [64]. It is thought that psyllium can significantly reduce cardiovascular risk factors including blood pressure, cholesterol levels, and blood glucose level [65]. In one study, supplementation with 3.4 g of psyllium per day for eight weeks in men was reported to reduce total cholesterol and LDL-C levels [66]. Psyllium intake has been reported to reduce systolic blood pressure by a mean of 2.24 mmHg with no significant effect on diastolic blood pressure, making it effective in the management of hypertension [67]. It was also shown to reduce total cholesterol by 9.05 mg/dL and LDL-C by 8.55 mg/dL [65]. These changes in lipid profiles suggest that psyllium may serve as an effective dietary intervention for hyperlipidemia, which is an important risk factor for cardiovascular diseases [13]. Psyllium was shown to increase the antioxidant capacity of the blood and reduce oxidative stress markers such as cardiac malondialdehyde [68]. It was also found to regulate inflammatory pathways that support cardiovascular health [13].

According to a meta-analysis study, psyllium achieved a mean reduction of 2.04 mmHg in systolic blood pressure [69], while other meta-analyses found that *Plantago ovata* consumption reduced LDL-C and triglyceride levels [64] and provided a reduction in LDL-C equivalent to that achieved by doubling the dose of statin while reducing the side effects caused by high-dose intake of statin group drugs [70]. Psyllium husk ethanol extract was shown to reduce total cholesterol, triglyceride, malondialdehyde, and atherogenic index values in hyperlipidemic rats; to increase superoxide dismutase and catalase levels [71]. In another study, male rats were fed a high-cholesterol diet for 8 weeks and 2.5% and 5% psyllium seed powder supplements were administered, and it was reported that the intervention significantly impacted lipid profiles while significantly increasing HDL-C [72]. A review study concluded that psyllium intake could significantly improve blood pressure levels and a dose of 10.5 g/day for 6 months could provide significant reductions in systolic and diastolic blood pressure [13].

Although numerous meta-analyses and clinical studies have reported that psyllium lowers LDL-C and systolic blood pressure levels, some studies have found no significant effects on HDL-C and diastolic blood pressure levels. The inconsistencies observed across studies may be attributed to differences in participants’ individual characteristics, variations in psyllium dose and form, and the duration of the intervention.

Although psyllium appears promising in improving cardiovascular health, individual differences in response to dietary interventions need to be considered. This suggests that psyllium should be considered not as a standalone treatment but as part of a comprehensive lifestyle approach. It would be helpful to investigate the long-term effects of psyllium on cardiovascular health and optimal dosages in more detail in future studies.

### Gastrointestinal System Diseases

Psyllium is known to have beneficial effects on gastrointestinal diseases. It may be used as a laxative to relieve constipation, as well as to help manage diarrhea and IBS symptoms, and it may have positive effects on gastrointestinal symptoms due to its high water absorption capacity and resistance to fermentation [73]. However, individual patient responses and possible interactions with other dietary fibers should be taken into account. When psyllium is combined with inulin, it reduces intestinal gas production but does not directly reduce fermentation [74].

Nonalcoholic fatty liver disease (NAFLD) increases in parallel with obesity and causes an increase in the risk of cirrhosis and liver cancer. Nutrition plays a very important role in chronic liver diseases such as NAFLD [75]. It is assumed that dietary fiber reduces the risk of NAFLD through its effects on obesity [76]. In a meta-analysis study, it was stated that 10–16 g/day fiber consumption for 10–12 weeks, particularly including psyllium, inulin, basil (*Ocimum basilicum*), and oligofructose, reduced BMI, alanine aminotransferase (ALT) and aspartate aminotransferase (AST) levels, HOMA-IR, and fasting insulin providing beneficial effects for NAFLD patients [77]. Another study reported that psyllium reduced hepatic lipogenesis [78]. The effectiveness of psyllium husk and that of weight loss medication were compared in mice fed a high-fat diet. In that study, psyllium husk had a better effect on serum and liver cholesterol and triglyceride levels and provided better results than weight loss drugs in the treatment of NAFLD. In contrast, weight loss drugs had a better effect on body weight loss, and both interventions exerted similar effects on body fat ratio [79]. Psyllium husk mucilage (100 mg/kg) was also found to reduce the harmful effects of carbon tetrachloride (CCl4) in rat liver and TNF-α expression in rats [80]. It was determined that in rats given fiber supplementation (psyllium, cellulose, pectin, and inulin) in addition to a diet enriched with copper nanoparticles, reductions of fat, cholesterol, and triglyceride levels in the liver were achieved together with reduced cyclooxygenase-2 (*COX-2*), peroxisome proliferator-activated receptor gamma (*PPAR-γ*), and sterol regulatory element-binding protein 1 (*SREBP-1*) gene expression and liver inflammation [81]. Psyllium (100 mg/kg) was also found to reduce hepatic inflammation and alcohol absorption in mice given a single high alcohol dose (4 g/kg), and it may have protective properties against liver damage [82].

Dietary modifications can alleviate the symptoms of gastroesophageal reflux disease, and increasing the intake of dietary fiber is among these modifications [83]. A meta-analysis study reported that psyllium can reduce gastroesophageal reflux symptoms [84]. Another study found that 5 g of psyllium supplementation administered to gastroesophageal reflux patients with low fiber intake reduced heartburn and gastroesophageal reflux disease questionnaire (GERD-Q) scores and increased resting lower esophageal sphincter pressure [85]. A similar study concluded that psyllium supplementation reduced esophageal motility from 71.4% to 14.3% [86]. It was also determined that psyllium seed extract (400 mg/kg) reduced ulcer index values and lipid peroxidation in rats, protected the integrity of the gastric mucosa, and increased the levels of antioxidant enzymes such as catalase, glutathione, and superoxide dismutase [87].

Soluble fiber is not recommended for gastroparesis patients as it can exacerbate symptoms such as nausea, abdominal pain, bloating, and vomiting. However, low-viscosity soluble fiber can be tolerated by gastroparesis patients [88]. It was reported that psyllium husk regulates blood glucose in gastroparesis patients and does not have a significant effect on its passage through the gastrointestinal system [89].

Low fiber consumption may be a risk factor for gallstones, and adequate fiber intake may have a protective effect against them [90]. In a study conducted on obese individuals, supplementation with psyllium (15 g/day) alongside a low-energy diet was found to have a similar effect to ursodeoxycholic acid in preventing gallstone formation [91]. In a study conducted on mice, it was reported that psyllium induced the expression of genes regulating bile acid secretion, activated the farnesoid X receptor that suppresses proinflammatory signals, and exerted a protective effect against colitis by altering bile acid metabolism [92]. Another study found that a 1:2 ratio of whey protein/psyllium tripled the binding ability of bile acid compared to psyllium alone [93].

Irritable bowel syndrome (IBS) is a chronic condition accompanied by abdominal pain and disrupted bowel movements [94]. A systematic review reported that psyllium can reduce IBS symptom [95]. It was also found that psyllium reduced the severity of IBS in pediatric IBS patients and may have positive effects on treatment [96]. Psyllium was shown to reduce pain attacks in children with IBS and functional abdominal pain disorder [97]. Another study demonstrated that psyllium reduced the colon gas caused by inulin in individuals with IBS and increased the tolerability of prebiotics in that patient population [74]. Low intake levels of vegetables, fruits, and fiber are among the factors that cause IBD. Dietary fiber has a positive effect on IBD by reducing inflammation and protecting the intestinal epithelial barrier [98]. However, a systematic review reported conflicting results on the effects of psyllium on IBD symptoms [99]. In one study, it was determined that *claudin 2*, *claudin 3*, *claudin 8*, and *occludin* gene expression levels were higher among mice with dextran sodium sulfate-induced colitis given psyllium compared to a control group, and the authors concluded that psyllium may have protective effects against colitis [100]. In another study investigating the effects of psyllium, cellulose, and inulin on the microbiota in individuals with IBD, it was reported that there were differences between participants in the interactions of psyllium and inulin with the microbiota and that the administered treatment reduced proinflammation [101].

In a meta-analysis study evaluating the effect of adding different dietary fibers to enteral nutrition on diarrhea, the effect of psyllium on diarrhea was found to be unclear [102]. Another study evaluated the effectiveness of a low fermentable oligosaccharides, disaccharides, monosaccharides, and polyols (FODMAP) diet or psyllium at 6 g/day for 4 weeks in adults with episodes of fecal incontinence. Both interventions were found to reduce episodes of fecal incontinence in individuals with loose stools [103].

Dietary fiber also may play an effective role in the treatment of chronic constipation [104]. Psyllium may have a positive effect on constipation by increasing bowel movements and stool water, facilitating defecation, and altering the colonic content [105, 106]. In a systematic review, it was stated that psyllium increased stool water content and stool softness in individuals with chronic idiopathic constipation [107]. A meta-analysis study reported that psyllium supplementation at a dose of > 10 g/day for at least 4 weeks was the most effective method for treating constipation [108]. One study concluded that psyllium increased bowel movements and reduced constipation symptoms in individuals with chronic constipation [109]. Similarly, in another study, psyllium was shown to increase stool water in individuals with constipation [105]. Yang et al. [110] reported that psyllium may reduce constipation symptoms. However, it was found that agave fructans and *Plantago psyllium* did not have an effect on intestinal transit time in individuals with functional constipation [111]. Other researchers demonstrated that psyllium (3.5 g) and lactitol (10 g) supplementation for 4 weeks increased intestinal motility in individuals with constipation, but the difference compared to the placebo group was not significant [112]. In a study conducted by Xu et al. [113] it was found that the administration of psyllium and *Ligilactobacillus salivarius* Li01 to mice increased the water content of their feces, improved the rate of gastrointestinal transit, and provided protection against constipation. Psyllium may help regulate intestinal function and may be used as a laxative in cases of constipation [32]. Although psyllium has many health benefits, it is necessary to take into account individual dietary requirements and possible side effects; in particular, excessive intake can lead to gastrointestinal symptoms.

### Cancer

It is predicted that there will be 35 million new cancer cases worldwide in 2050 [114]. Psyllium may help protect the intestinal structure and may inhibit carcinogenesis by reducing the levels of inflammatory mediators such as TNF-α and NO. It also may increase stool volume, may accelerate intestinal transit, and may reduce the concentration of carcinogens. Psyllium may reduce cell proliferation in the distal colon via butyric acid and may help prevent the transformation of colonic epithelial cells into cancer [115].

Psyllium has been used to develop hydrogels for the sustained delivery of anticancer drugs. Hydrogels offer valuable properties such as mucoadhesion, non-hemolytic structures, and antioxidant activity, indicating that they can be used in the treatment of cancers of the gastrointestinal system [33]. In an animal study, the combination of psyllium husk, guar gum, and wheat bran was found to increase hydrated feces mass and may reduce constipation, indirectly lowering the risk of colorectal cancer [116]. Psyllium capecitabine-loaded core-shell nanoparticles have shown high cytotoxicity against the HCT-15 colon cancer cell line and may exert significant anticancer activities [117]. In a study investigating the effects of psyllium, pectin, inulin, and cellulose fiber on high-fructose diet-induced chronic colitis and colitis-associated colorectal neoplasia in male C57BL/6J mice, it was determined that these dietary fiber sources significantly suppressed the increase in colorectal tumor incidence, number, and size [118]. Furthermore, psyllium, cellulose, and inulin pulp delayed tumor growth and significantly reduced tumor size in C57BL/6 mice with bladder cancer [119]. A bionanogel combined with caffeic acid and IR-820 carrying a nanocomplex comprising psyllium mucilage polysaccharide and bacterial gellan gum was described as a potential new approach for drug delivery in the treatment of epidermoid tumors [120]. It was found that high-fructose diets increased tumor formation in cases of colitis in mice but psyllium supplementation exerted protective effects against tumor formation [121]. Furthermore, the addition of psyllium supplementation was shown to increase the treatment efficacy of the MR-HIFU method used for uterine myoma [122]. Although psyllium thus shows promise in cancer treatment, it is important to consider the broader context of its use. The majority of existing studies on the anticancer effects of psyllium consist of preclinical research conducted in vitro and in animal models. More research is needed to fully understand and optimize the contributions of psyllium in cancer treatment. The efficacy of psyllium in clinical settings needs to be evaluated comprehensively.

### Other Diseases

Evidence on the effects of psyllium on kidney disease is quite limited, and the available data are mostly derived from animal models. One previous study reported that psyllium seed husk reduced blood urea nitrogen and serum creatinine levels in the 5/6 nephrectomy rat model, alleviated renal tubular interstitial damage, downregulated indoxyl sulfate, lowered the levels of interleukin-6 (IL-6) and interleukin-1 (IL-1), regulated the intestinal microbiota, and may have therapeutic effects in the treatment of chronic kidney disease [123].

**Fig. 2:**
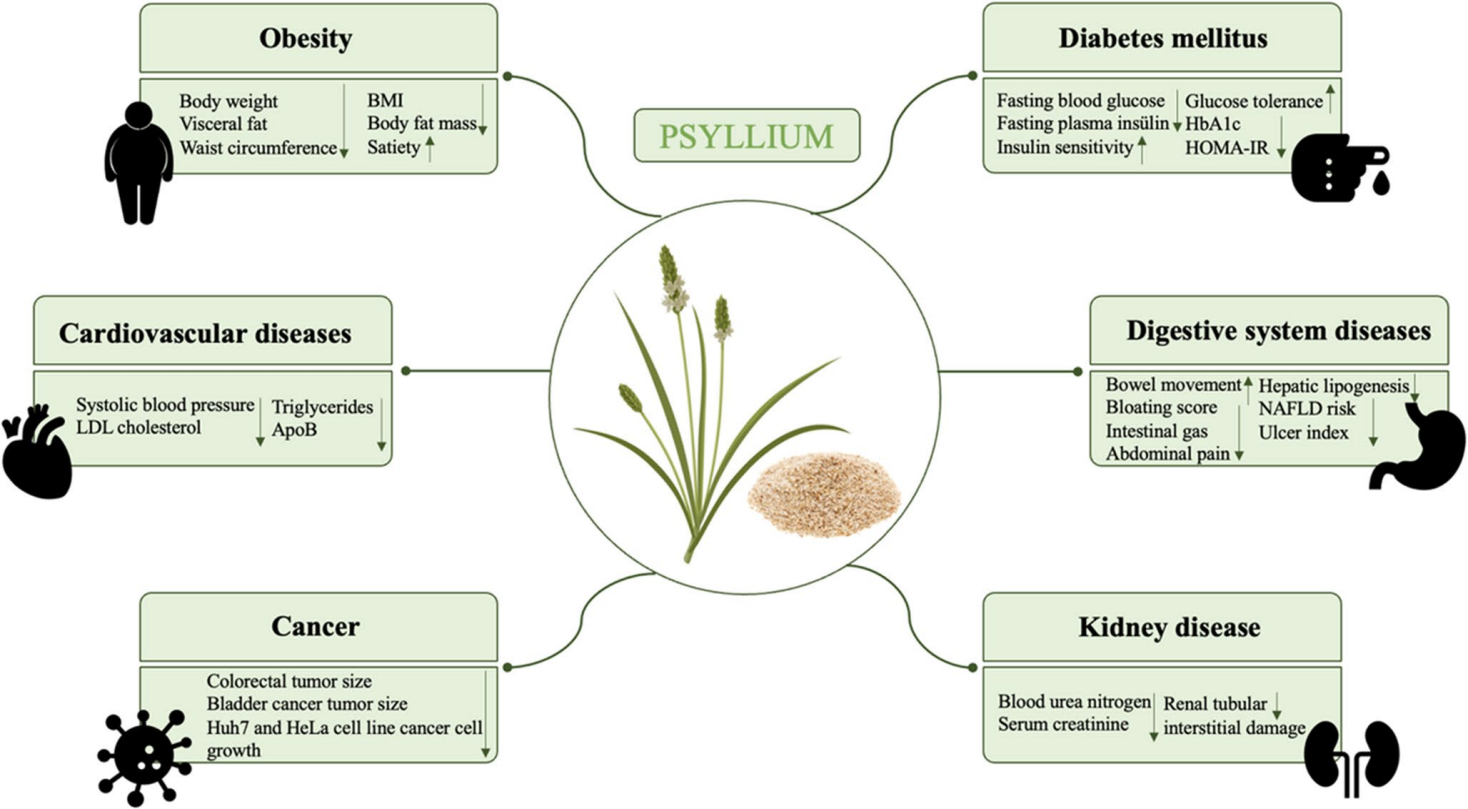
Use of psyllium in food products

Burns constitute the fourth most common type of injury worldwide. Moist wounds cause microorganisms to multiply, leading to infection [124]. Applied with the aim of reducing the moisture in burn wounds, *Plantago ovata* mucilage was also found to significantly reduce wound size [125]. The health effects of psyllium are shown in Fig. 2.

Meta-analysis, in vivo, in vitro, animal, and human studies conducted to evaluate the health effects of psyllium are summarized in Table 2.

**Table 2.**
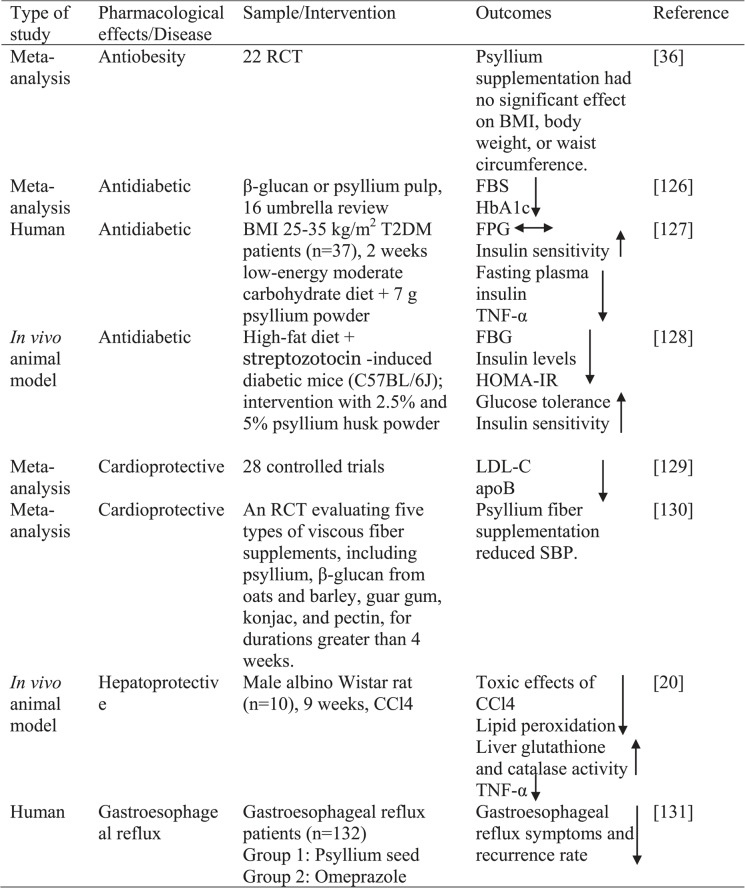

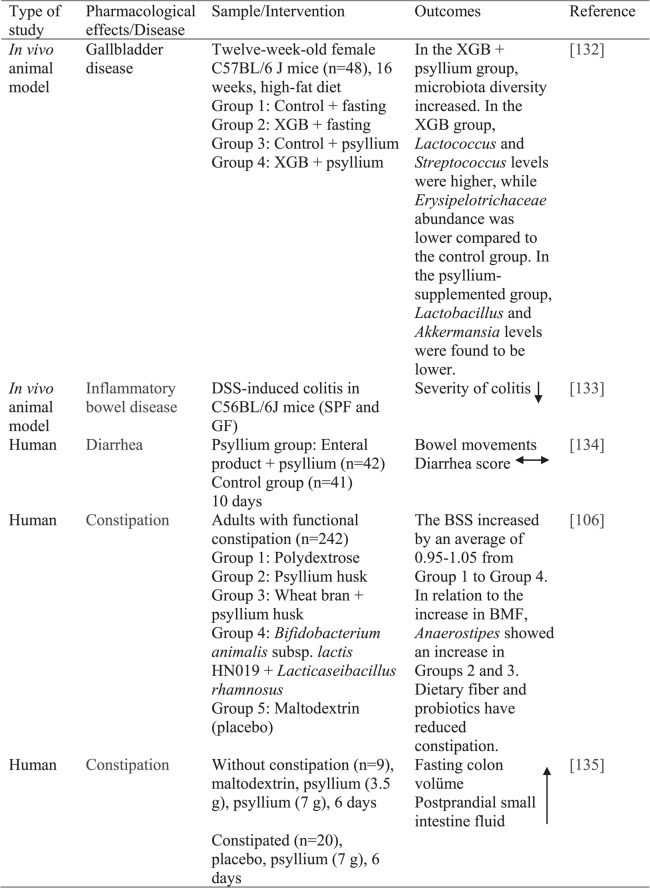

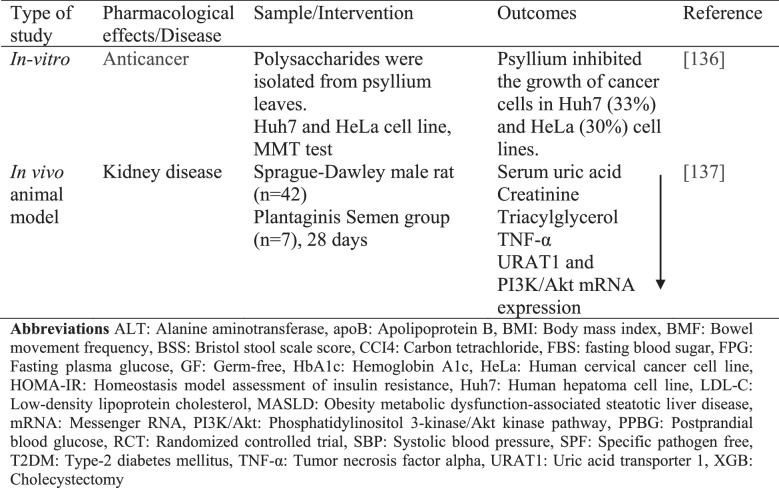
Studies on the effects of psyllium on health

*ALT* Alanine aminotransferase, *apoB* Apolipoprotein B, *BMI* Body mass index, *BMF* Bowel movement frequency, *BSS* Bristol stool scale score,*CCI4* Carbon tetrachloride, *FBS* fasting blood sugar, *FPG* Fasting plasma glucose, *GF* Germ-free, *HbA1c* Hemoglobin A1c, *HeLa* Human cervical cancer cell line, *HOMA-IR* Homeostasis model assessment of insulin resistance, *Huh7* Human hepatoma cell line, *LDL-C* Low-density lipoprotein cholesterol, *MASLD* Obesity metabolic dysfunction-associated steatotic liver disease, *mRNA* Messenger RNA,*PI3K/Akt* Phosphatidylinositol 3-kinase/Akt kinase pathway, *PPBG* Postprandial blood glucose, *RCT* Randomized controlled trial, *SBP* Systolic blood pressure, *SPF* Specific pathogen free, *T2DM* Type-2 diabetes mellitus, *TNF-α* Tumor necrosis factor alpha, *URAT1* Uric acid transporter 1, *XGB* Cholecystectomy

### Safe Intake Level and Toxic Effects

The European Food Safety Authority has reported that the amount of psyllium seed husk providing a feeling of satiety and helping to control body weight is 1–3 g/day [138]. Other important aspects of the safety of psyllium supplementation are the purity and degree of processing of the psyllium. The US Food and Drug Administration has stated that psyllium with purity of at least 95% at 10.2 g/day is safe and offers therapeutic effects. The literature suggests that the appropriate dosage for therapeutic effects is 3.4–5 g three times a day before main meals, totaling 10–15 g/day. In cases other than constipation, it is recommended to start at 3.4 g/day and increase the dose every week to reach 10.2 g/day [115]. Researchers report that psyllium supplementation at 10 g does not cause any side effects in individuals with type 2 diabetes [54]. It has also been stated that psyllium husk does not cause side effects in women with chronic constipation [110]. In addition to its beneficial effects on health, some common and, though rare, serious adverse effects of psyllium have also been observed [11]. In a case report, the use of psyllium for the treatment of constipation was reported to cause severe dizziness, wheezing, and loss of consciousness [139]. High doses of psyllium can also lead to increased intestinal gas production and abdominal bloating, which are among the most commonly reported mild side effects [134]. Serious adverse effects of psyllium are generally rare; however, in some individuals, it may cause allergic reactions, gastrointestinal symptoms, anaphylaxis, and skin reactions. It can act as a strong inhalant allergen and cause asthma symptoms [11]. In a case report, psyllium was observed to potentially exacerbate asthma symptoms [140], while another case report documented allergic rhinitis in a bakery worker due to exposure to a psyllium-containing product [141]. If sufficient fluids are not consumed while taking psyllium powder and the powder is not swallowed completely, it causes bloating of the throat and obstruction of the esophagus. Therefore, it is recommended to drink a glass of water with each dose of psyllium powder [115]. Several case reports have described intestinal obstruction associated with insufficient fluid intake during psyllium use [142, 143]. Thus, although psyllium has beneficial effects for many individuals, it is necessary to pay attention to its potential toxic effects, especially in individuals with gastrointestinal sensitivity [82]. Adequate fluid intake is essential when using psyllium, and this should be emphasized as a critical public health message to prevent gastrointestinal obstruction.

### Limitations and Strengths

A significant limitation of this review is that the types of psyllium (e.g., powder, shell, or seed) and the doses used in the studies in the literature differ. This makes it difficult to draw general conclusions. Furthermore, the fact that these studies were conducted among different populations also limits the generalizability of the results to some extent. There are differences in the methods and follow-up periods of the studies in the literature, and that prevents clear results from being obtained in the long term.

However, psyllium appears to have beneficial effects for many health problems, including obesity, cardiovascular diseases, diabetes mellitus, cancer, and gastrointestinal diseases. These positive effects highlight the multifaceted benefits that psyllium provides thanks to its soluble fiber contents and different biological mechanisms of action. This review of the literature has confirmed that psyllium is an effective ingredient with wide potential for use in the field of health as both a complementary and a preventive compound.

Future studies should compare the effects of different psyllium types and doses with more homogeneous study designs, taking into account differences across populations. With such planning, the effects of psyllium on health can be revealed more clearly and more effective usage strategies can be developed in clinical practice.

### Conclusion and Future Perspective

Psyllium may have beneficial effects on metabolic, cardiovascular, gastrointestinal, renal and inflammatory pathways. While the current scientific evidence highlights its benefits, variations in formulation, dosage and study duration limit the comparability of studies. Future research should prioritise standardising psyllium forms and dosages, and conducting long-term randomised controlled trials examining outcomes such as glycaemic control, lipid metabolism, blood pressure, NAFLD, and gastrointestinal function. Mechanistic studies focusing on bile acid dynamics, appetite-related hormones and microbiota–host interactions will further clarify the underlying pathways of psyllium’s effects. Additionally, research related to functional food applications should address parameters such as texture, sensory acceptability, stability and real-world effectiveness. Overall, psyllium is a promising functional ingredient with potential applications in health and industry, and more comprehensive research will facilitate its optimal use.

## Key References

- Gholami Z, Clark CCT, Paknahad Z. The effect of psyllium on fasting blood sugar, HbA1c, HOMA IR, and insulin control: A GRADE-assessed systematic review and meta-analysis of randomized controlled trials. BMC Endocr Disord. 2024;24(1):82. 10.1186/s12902-024-01608-2.
  - **○ **This article is a systematic review and meta-analysis of randomized controlled trials investigating the effects of psyllium on fasting blood sugar, HbA1c, HOMA-IR, and insulin levels. It was shown that psyllium significantly reduces fasting blood sugar, HbA1c, and HOMA-IR levels, but has no significant effect on insulin levels.
- Gholami Z, Paknahad Z. The effect of psyllium consumption on blood pressure: Systematic review and dose-response meta-analysis of randomized controlled trials. Food Sci Nutr. 2024;12(10):7075–87. 10.1002/fsn3.3863.
  - **○ **This article is a systematic review and meta-analysis of randomized controlled trials investigating the effects of psyllium on blood pressure. It was reported that psyllium significantly reduces systolic blood pressure, while it has no significant effect on diastolic blood pressure.
- Xu L, Qiu B, Ba F, Zhang S, Han S, Chen H, et al. Synergistic effects of *Ligilactobacillus salivarius* Li01 and psyllium husk prevent mice from developing loperamide-induced constipation. Food Funct. 2024;15(24):11934–48. 10.1039/d4fo04444d.
  - **○ **This article is an animal study investigating the effects of the combined use of psyllium and *Ligilactobacillus salivarius* Li01 on constipation. It was shown that this combination provides protection against constipation by improving gastrointestinal transit rate and increasing fecal water content.

## Supplementary Information

Below is the link to the electronic supplementary material.

ESM 1 (PDF 380 KB)

## Data Availability

All data needed to evaluate the conclusions in this article are included in the article. Additional data related to this article may be requested from the authors.

## Abbreviations

ALT: Alanine aminotransferase
AST: Aspartate aminotransferase
BMI: Body mass index
COX-2: Cyclooxygenase-2
FODMAP: Fermentable oligosaccharides, disaccharides, monosaccharides, and polyols
FTO: Fat mass and obesity-associated gene
GERD-Q: Gastroesophageal reflux disease questionnaire
GLP-1: Glucagon-like peptide-1
HbA1c: Hemoglobin A1c
HDL-C: High-density lipoprotein cholesterol
HOMA-IR: Homeostasis model assessment of insulin resistance
IBD: Inflammatory bowel diseases
IBS: Irritable bowel syndrome
IL-1: Interleukin-1
IL-6: Interleukin-6
LDL-C: Low-density lipoprotein cholesterol
LEP: Leptin
LEPR: Leptin receptor
MC4R: Melanocortin 4 receptor
NAFLD: Non-alcoholic fatty liver disease
NO: Nitric oxide
PPAR-γ: Peroxisome proliferator-activated receptor gamma
RELMβ: Resistin-like beta
SPRR2A: Small proline-rich protein 2 A
SREBP-1: Sterol regulatory element-binding protein 1
TNF-α: Tumor necrosis factor alpha
