# Supplementary material for: From Husks and Seeds to Health: an Inevitable Outcome Rather than a Fluke
Source: Curr Nutr Rep. 2026 Feb 26;15(1):15. doi: 10.1007/s13668-025-00722-4 (PMC12935724; doi:10.1007/s13668-025-00722-4)
Supplement: Supplementary file 1 — (PDF 380 KB) [file 13668_2025_722_MOESM1_ESM.pdf]

Certificate of Editing

This is to certify that the manuscript

From Husks and Seeds to Health: An Inevitable Outcome Rather Than a Fluke

By the author

Prof. Dr. Nevin ŞANLIER

Has been edited for English language and grammar

as well as

scientific content and formatting

March 10, 2025

Date

Leslie Demir

Academic copy editor and proofreader

leslie.demir@gmail.com
